# Do Walking Distance and Time Away from the Paddock Influence Daily Behaviour Patterns and Milk Yield of Grazing Dairy Cows?

**DOI:** 10.3390/ani11102903

**Published:** 2021-10-07

**Authors:** Heather W. Neave, J. Paul Edwards, Helen Thoday, Katie Saunders, Gosia Zobel, James R. Webster

**Affiliations:** 1 Animal Behaviour and Welfare Team, AgResearch Ltd., Ruakura Research Centre, 10 Bisley Road, Private Bag 3123, Hamilton 3214, New Zealand; gosia.zobel@agresearch.co.nz; 2 DairyNZ Ltd., P.O. Box 85066, Lincoln 7647, New Zealand; paul.edwards@dairynz.co.nz (J.P.E.); katie.saunders@dairynz.co.nz (K.S.); 3 DairyNZ Ltd., Private Bag 3221, Hamilton 3240, New Zealand; helen.thoday@dairynz.co.nz; 4 Animal Ethics Office, AgResearch Ltd., Ruakura Research Centre, 10 Bisley Road, Private Bag 3123, Hamilton 3214, New Zealand; jim.webster@agresearch.co.nz

**Keywords:** milking time, time budget, animal welfare

## Abstract

**Simple Summary:**

Dairy cows managed on pasture may need to walk several kilometres to reach the milking parlour to be milked and, thus, may spend an extended time away from the paddock without access to pasture. While cows have a diverse behavioural repertoire, at a minimum they must graze, ruminate and lie down; therefore, time spent away from the paddock will affect how these behavioural needs are met. We investigated how walking distance to the milking parlour and total time spent away from the paddock affected daily grazing, ruminating and lying behaviours of dairy cattle managed in three groups. The devices automatically monitored cow behaviour and the time spent walking and waiting outside the paddock. We showed that cows spent more time grazing and less time ruminating on days with longer walking distances, which may, in part, be due to greater energy expenditure resultant of walking. When cows spent more time away from the paddock, they had a reduced lying time, and the cows in one of the groups produced less milk. Thus, limiting the walking distance and time spent away from the paddock are two factors that could provide greater opportunities for cows to engage in daily behavioural patterns that meet individual needs.

**Abstract:**

In pasture-based systems, cows may spend several hours away from the paddock and may also walk several kilometres to meet daily milking requirements; this could lead cows to experience time constraints for grazing, ruminating and lying time in the paddock. This study investigated how walking distance and time spent away from the paddock affected daily behavioural patterns (i.e., grazing, ruminating and lying time) and milk yield. Dairy cows were managed in three rotationally grazed groups (*n* = 29 cows each) on the same farm and milked twice daily. A triaxial ear tag accelerometer on each cow recorded daily duration of grazing and ruminating, and a leg-based accelerometer recorded the daily lying time, for 13 days. GPS collars on four cows per group recorded the daily walking distance and total time away from the paddock for the group. A mixed repeated measures model tested how time off-paddock and walking distance affected the daily behavioural patterns; age, breed, milk yield and maximum ambient temperature were used as covariates with group as the observational unit. A second similar model tested how these factors affected milk yield. Walking distance and time spent away from the paddock were not correlated. When daily walking distance increased (to a maximum of 4 km/d), cows spent more time grazing and less time ruminating, but lying time was not affected. This result may, in part, be related to the greater energy expenditure demands for walking longer distances and milk production. When time away from the paddock increased (to a maximum of 4 h/d), cows spent less time lying, but grazing and ruminating times were not affected. Milk yield was not affected by walking distance, but one of the groups experienced a lower milk yield when time away from the paddock was increased. This result suggests that, for some cows, lying times may be shorter when experiencing a longer time away from the paddock, which may also affect milk yield. Overall, this study indicates that paddock behaviours are associated with walking distance to the milking parlour and time spent away from the paddock. Efforts to reduce walking distance and time spent away from the paddock are likely to provide cows with greater opportunity to engage in daily behaviours in the paddock that meet their needs and maintain their milk yield.

## 1. Introduction

Pasture-based dairy farms may require cows to walk several kilometres to and from the milking parlour, resulting in several hours that could be spent away from the paddock (i.e., in the outdoor area where cows are kept for grazing). For instance, a survey of Australian dairy farmers reported that, for herds of over 300 cows, the walking distance was more than 1.9 km from the farthest paddock to the milking parlour (resulting in up to 8 km of walking per day when milked twice daily), and the longest times out of the paddock were over 3.5 h per milking [1]). A follow-up study of 10 commercial herds in Australia found that paddocks could be up to 3.5 km away from the milking parlour, amounting to over 6 h away from the paddock each day [2]. These distances and waiting times in summer months can contribute to heat stress, especially with increased body temperatures during and after walking [3]. In such circumstances, there is also less time available to perform other important daily behaviours in the paddock, such as grazing and lying. Under time constraints, dairy cows will prioritise lying time by decreasing time spent eating and socializing, indicating lying behaviour is important to cows and thus serves as a welfare indicator [4]. Lying deprivation induces stress responses through increased activity of the hypothalamic–pituitary–adrenal axis [5,6], and cows show signs of restlessness and discomfort when deprived of lying for 2 to 4 h [7]. In addition, milk production may decrease if the feeding and lying time are restricted, even for short periods [8]. Thus, longer walking distances and waiting times on pasture-based farms may negatively impact dairy cow welfare. 

Pastured dairy cows milked twice daily typically spend between 9 and 11 h/d lying [2,9,10,11,12], between 7 and 9 h/d grazing [13,14,15] and between 6 and 8 h/d ruminating [16], with periods of ruminating occurring while lying down [17]; however, these time budgets are based on the time cows spend in the paddock. Indeed, cows in large pasture-based herds (over 500 cows) decreased their lying time when the waiting time at the milking parlour increased (approximately 14 min less lying time for every 1 h increase in waiting time [2]). Longer waiting times, in combination with longer walking distances, can lead to a greater total time spent away from the paddock, particularly since some cows are consistently milked last in the milking order [18]. Individual cows are known to respond differently to various challenges [19]; thus, additional waiting times or energy expenditures due to walking longer distances may be compensated for in different ways by individual cows. For instance, Beggs et al. [2] found that the waiting time at the milking parlour affected the lying time, but it only explained 14% of the individual variation in the lying time. This suggests that other factors may be affecting their daily behaviour patterns. It is currently unknown how other daily behaviour patterns like grazing and ruminating time are impacted by increased walking distances and time away from the paddock in grazing dairy cattle. 

The primary objective of this study was to determine if daily walking distance and total time away from the paddock were associated with daily behaviour patterns (grazing, lying and ruminating time) of dairy cattle managed on pasture. A secondary objective was to determine if these factors were also associated with daily milk production.

## 2. Materials and Methods

All procedures were approved by the Ruakura Animal Ethics Committee in Hamilton, New Zealand (#14876). The study was conducted from 14 November to 30 November 2019 at the Lincoln University Research Dairy Farm in Lincoln, New Zealand (−43.639 latitude, 172.456 longitude). During the study period, average daily ambient temperature was 14.5 ± 3.3 °C (daily average maximum 20.7 ± 4.3 °C; daily average minimum 10.1 ± 3.5 °C), with the hottest day reaching 29.9 °C. Rainfall occurred on three days of the study period (4.8 mm, 7.6 mm and 14.8 mm total daily rainfall) (New Zealand National Climate Database, station #17603).

### 2.1. Animal Management and Study Design

Three groups of 29 cows each (total 87 cows) were enrolled in the study and managed on a single farm; these animals were part of a concurrent study examining the effect of milking frequency (P. Edwards, pers. comm.). Multiparous cows were blocked for age, genetic merit, expected calving date, body condition score and liveweight at the end of the previous lactation, as well as the previous lactation days in milk, milk weight and milk solid yields and were randomly allocated to each groups. Primiparous cows were blocked by liveweight and genetic merit. All cows were in peak lactation (80 ± 19 d postpartum) due to the seasonal block calving system used in New Zealand. Cows were aged (mean ± SD) 3.7 ± 2.0 years (range: 2–12 years) and milked twice per day at 0500h and 1500h, with a milk yield of 22.4 ± 4.9 kg/d (range: 8.6–37.4 kg/d). All cows were crossbred (Friesian × Jersey), with 48 predominantly Friesian: 8 predominantly Jersey, and 31 an equal split of Friesian and Jersey. Each group was brought to the milking parlour close to their milking time to minimise the waiting time, and cows were milked together in a 24-stall (double 12 aside) herringbone parlour; group order of milking was maintained. All cows were returned to the paddock together as a group; therefore, the daily time spent away from the paddock was the same for all cows of a given group, and the waiting time at the milking parlour was similar for all cows (maximum 15 min before and 15 min after milking). Each group was on a grazing rotation of 10 0.75-ha paddocks, resulting in a total stocking rate of 3.9 cows/ha. A 20-d paddock rotation length was targeted where each group grazed a new paddock every 2 d; thus, each group had different walking distances to the milking parlour every second day. Prior to the experiment, the paddocks had been blocked for previous management and distance to the milking parlour and randomly assigned to a group. The paddocks were divided into four strips, with one strip grazed after each milking. The daily pasture dry matter allocation was targeted at 19 to 20 kg DM/d per cow (perennial ryegrass–white clover pasture, consisting of 80% ryegrass, 4% clover, 4% weeds, 11% dead and 1% other grasses, with 19 ± 0.9% DM, 17 ± 3.2% DM CP, 46 ± 2.8% DM NDF and 24 ± 1.3% DM ADF, analysed at Lincoln University (Lincoln, New Zealand) using near-infrared spectrophotometry). Two cows from Group 2 received a 0.5-kg barley supplement at each milking (1 kg/d) because of body conditions; this was received throughout the study for one cow, and in the last 2 d of the study for the second cow. 

### 2.2. Behaviour Data Collection

Daily lying times were monitored with pedometers (IceQube data loggers, Ice Robotics Ltd., Roslin, UK) previously validated in grazing dairy cattle by Reference [20]. Daily grazing and ruminating times were monitored with electronic ear tags (CowManager, CowManager SensOor, Agis Automatisering BV, Harmelen, The Netherlands) previously validated in grazing dairy cattle by Reference [21]. For all enrolled cows, the pedometers were attached to the right hind leg while in the milking parlour, and the electronic ear tags were attached to the existing radio frequency ID ear tag while the cows were restrained. The electronic ear tags automatically downloaded data to a server when the cow entered the milking parlour twice per day, and the pedometer loggers stored data on the logger until weekly manual downloading in the milking parlour. Time spent out of the paddock (hereafter referred to as “time off-paddock”) and walking distance were measured using GPS devices (Oyster2, Digital Matter, Subiaco, Australia) attached to the neck collars of four cows per group. These variables were recorded each day and were automatically uploaded to an online server. Time off-paddock was inclusive of walking time to and from the milking parlour, waiting time in the yards and milking time. Milk meters (DeLaval, Tumba, Sweden) automatically recorded the milk yield at each milking for each cow. 

The cows were monitored for paddock behaviours, walking distance and time off-paddock for a total of 16 d. 

### 2.3. Data and Statistical Analysis

All statistical analyses were performed using SAS (version 9.4; SAS Institute Inc., Cary, NC, USA). Data from the electronic ear tag devices (reported as total min/h for each behaviour) were summarised into daily durations of grazing and ruminating for each cow. All occurrences of rumination while standing, lying or walking were considered in the daily total. Grazing was not possible while walking to and from the milking parlour. Daily data from the automated behaviour devices were examined for biologically unlikely measurements (such as zero, very high or very low (±3 SD beyond the mean) daily grazing, ruminating or lying times) and excluded from the analysis (2% of all data). The daily total walking distance and daily total time spent away from the paddock were calculated from the list of trips recorded by the GPS collars. A trip was recorded when the cow moved at least 100 m without stopping. Each recorded trip route was mapped online using the Digital Matter Telematics Guru platform software and verified that only trips to and from the milking parlour were considered in the calculation for the time off-paddock and daily walking distance. There were 13 d with complete trip recordings, and thus, these days were examined for the analyses described below.

Each group had 13 different walking distances and times off-paddock available for analysis (all cows within a group had the same distance and time off-paddock recorded on a given day). All behaviours, walking distances and time off-paddock measures were normally distributed (assessed using PROC UNIVARIATE and model residuals) and summarised into the daily duration of grazing, ruminating and lying; daily distance walked and daily time off-paddock for each group.

The daily walking distance and daily time spent off-paddock were tested in the same model for their effects on behavioural outcomes, after determining no autocorrelation using Pearson’s correlation and no differences in the mean values between groups using a *t*-test (see Section 3). Regression analyses were performed with the groups as the observational unit. To test the effects of daily walking distance and time off-paddock on the daily behaviour patterns of cows, repeated measures regression models (PROC MIXED) were applied for the time spent grazing, ruminating and lying outcome variables. Experimental day (d 1–13) was the repeated measure with a first-order autoregressive covariance structure based on the lowest Akaike information criterion (AIC). Group was a random effect, and daily milk yield and maximum daily temperature were included as fixed effects. Daily rainfall was also initially included as a fixed effect but was never significant (*p* > 0.20), so was removed from all models.

The effects of daily time off-paddock and daily walking distance on milk yield were analysed within group due to the differences in milk yields between groups (see Section 3). A similar model structure to that described above was used with the following exceptions: (1) a compound symmetry covariance structure was selected based on the lowest AIC; (2) daily grazing, ruminating and lying times were also included as fixed effects and (3) cow was the experimental unit. Time spent off-paddock and walking distance on a given day were expected to affect the next day’s milk yield, so the outcome variable was the change in milk yield.

Results were reported as significant at *p* ≤ 0.05, and test statistics were reported as F or *t* numerator degrees of freedom and denominator degrees of freedom. 

## 3. Results

**Figure 1:**
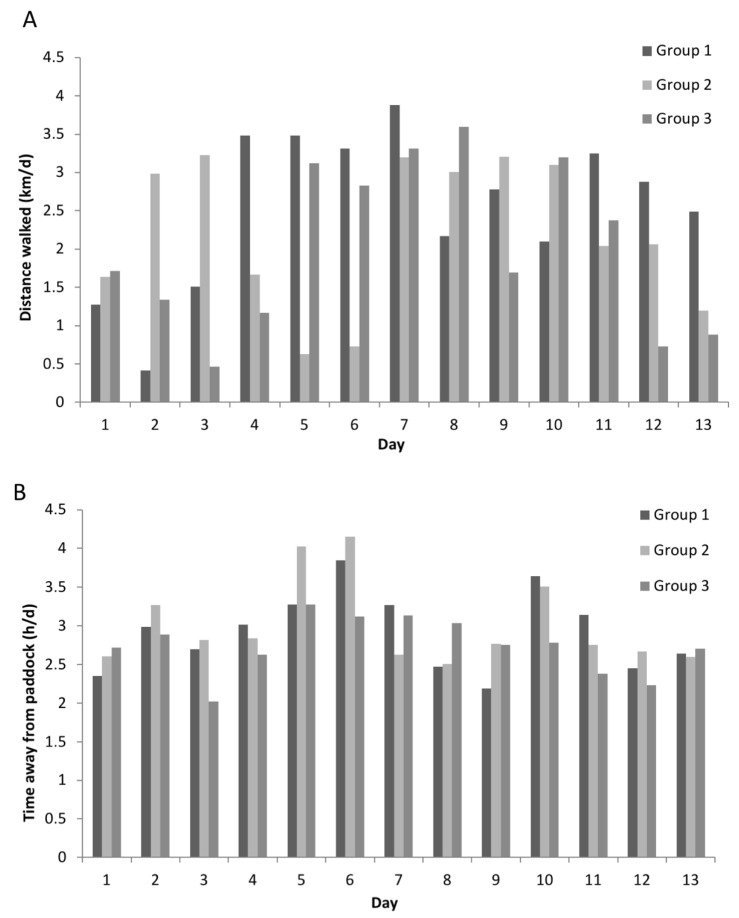
Daily average (**A**) walking distance to and from the milking parlour (km/d) and (**B**) time spent away from the paddock (h/d), inclusive of the walking time, waiting time before and after milking and milking time. Averages are for each of the 3 groups over the 13-d study period, measured using GPS collars (data from 4 cows/group).

The cows walked 2.5, 2.2 and 2.0 ± 1.1 km/d and spent 2.9, 3.0 and 2.7 ± 0.6 h/d away from the paddock (mean ± SD for Group 1, Group 2 and Group 3, respectively) (Figure 1). Walking distance and time spent away from the paddock were not correlated (r = 0.17; *p* = 0.31). The daily behaviour patterns (grazing, ruminating and lying time) of the cows in each group are reported in Table 1. There were no differences between the groups for walking distance, time spent away from the paddock or daily behaviours (F_2,36_ < 1.0; *p* > 0.37). 

**Figure 2:**
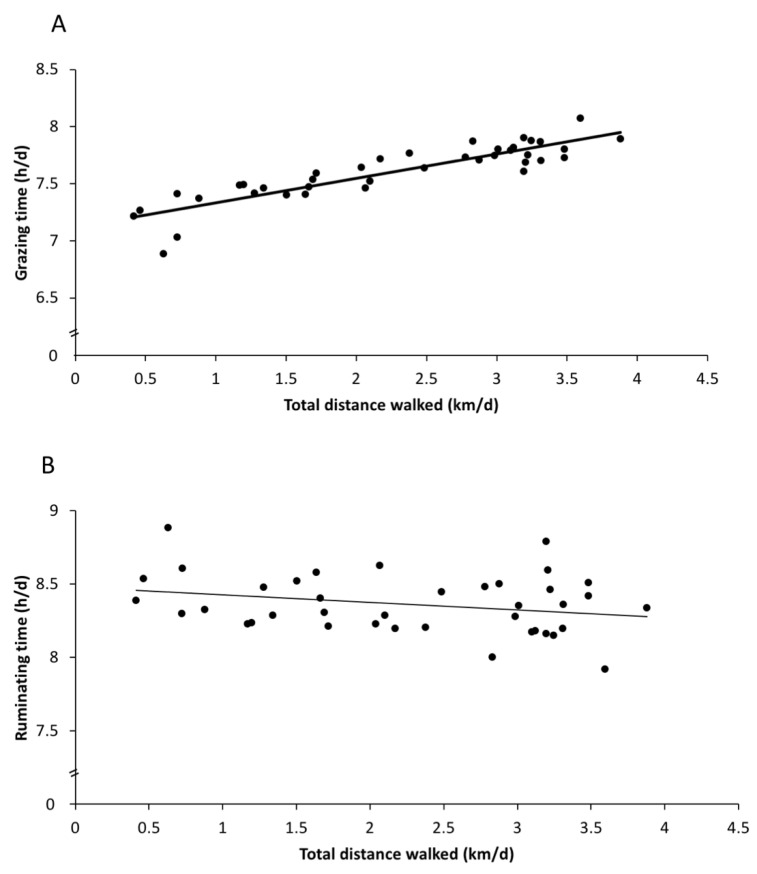
Regression analysis of the total distance walked against behaviours in the paddock. (**A**) Grazing time increased with the distance walked, and (**B**) ruminating time decreased with the distance walked. The predicted values from the regression models are plotted for each of the 13 days for the 3 groups.

**Figure 3:**
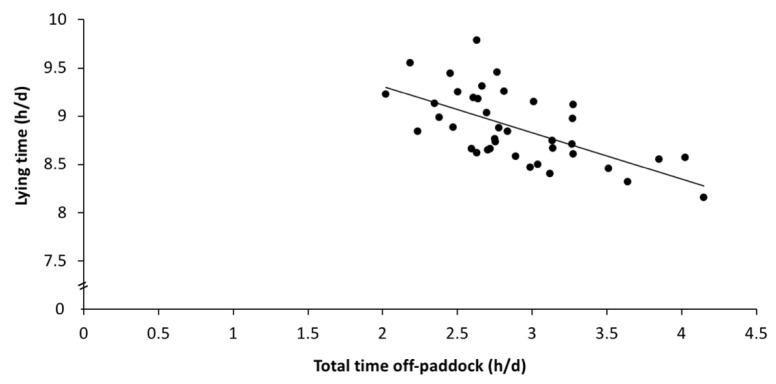
Regression analysis of the total time off-paddock against lying time in the paddock. The predicted values from the regression models are plotted for each of the 13 days for the 3 groups.

Large individual variabilities in the daily behaviour patterns were observed; for instance, some cows spent only 4 h grazing (while others spent up to 10 h grazing), and some cows were lying down for about 7 h (while others were lying down for over 11 h). Some of this individual variation could be explained by walking distance and time off-paddock. On days that cows walked greater distances, grazing time increased by about 14 min (Figure 2A; estimate: 13.9 ± 4.8 min/km walked; F_1,32_ = 8.3; *p* = 0.007), and ruminating time decreased by about 7 min for each additional km walked (Figure 2B; estimate: −6.8 ± 3.3 min/km; F_1,32_ = 4.1; *p* = 0.05), but there was no association with lying time (F_1,32_ = 0.68, *p* = 0.42). On days that cows spent more time off-paddock, lying time decreased by about 30 min for each additional hour spent off-paddock (Figure 3; estimate: −34.2 ± 14.2 min/h off-paddock; F_1,32_ = 5.9; *p* = 0.02), but there was no association with the grazing or ruminating times (F_1,32_ < 2.0, *p* > 0.16). 

The average daily milk yield over the study period was greater in Groups 1 and 2 (mean ± SE; 22.6 ± 0.18 and 22.9 ± 0.18 kg/d, respectively) compared with Group 3 (21.6 ± 0.18 kg/d; t_1,30_ > 4.0; *p* < 0.01). The change in the milk yield was not associated with daily walking distance for any of the groups (F_1,6_ < 1.5, *p* > 0.27). The change in the milk yield for Group 3 was negatively associated with time off-paddock (estimate: −1.3 ± 0.009 kg/d/h off-paddock; F_1,6_ = 6.7, *p* = 0.04), but this was not the case for the other two groups (F_1,6_ < 1.5, *p* > 0.27).

## 4. Discussion

Our study examined how two farm management factors, time spent away from the paddock and walking distance, affected the behaviour of dairy cattle managed on pasture. We found that daily lying time decreased with greater time spent away from the paddock but not with greater walking distance. In contrast, daily grazing and ruminating time were both affected by walking distance but not the time spent away from the paddock. These results suggest that the time spent away from the paddock and walking distance affected cows in different ways.

The daily behaviour patterns of dairy cows in this study were within the range of values previously reported for grazing time [13,14,15] and lying time [2,12,22,23,24] of pastured dairy cattle. The cows in this study spent, on average, more time ruminating than reported in other studies on pastured dairy cattle [16,25]. These differences may be due to dietary factors, such as the physical and chemical composition of available forage, but rumination time can also depend on the milk yield and parity of the cow (reviewed by Beauchemin [26]). There may also be differences in how collar-based and ear-based accelerometers record rumination times, so caution should be taken when comparing between studies. As expected, large individual variation in all recorded behaviours was observed, with some cows spending several hours less time engaged in grazing or lying than the group average. Our study found that farm and management factors, such as walking distance to the milking parlour and total time spent off-paddock (inclusive of the walking time, waiting and milking time at the parlour), explained some of these individual differences in daily behaviour patterns.

Walking distance and time away from the paddock were expected to affect the behaviour patterns of grazing dairy cattle. These two factors were not correlated; this was not unexpected, since it was observed that the milking duration each day was variable depending on the milker (i.e., milker speed impacted the return time to paddock more than distance). We found that, on days when cows walked greater distances to and from the milking parlour, grazing time increased and ruminating time decreased, with no effect on the milk yield. The increase in grazing time may be related to an increased appetite due to the greater energy expenditure required of walking longer distances while maintaining the cow’s physiological demands for milk production [27]. However, it is unlikely that the extra grazing time (of 15 min for each additional km walked) would be necessary to account for the additional energy needed for walking (0.14 kg DM/km [28]), given that one study estimated the intake rate of pastured cows to be 1.78 kg DM/h, equivalent to 0.45 kg DM/15 min [29]. Alternatively, increased grazing time can occur if the bite size is smaller, which may also lower rumination time due to shorter particle lengths and passage rates through the rumen [30]. However, it is unclear why walking distance might affect bite size. It is possible that other factors that were not measured in this study contributed to an increased grazing time on days with greater walking distances.

The cows in our study walked about 2 km/d on average, which is lower than the reported walking distances in pasture-based commercial herds. For instance, New Zealand farmers self-reported an average one-way distance of 1.9 km from the paddock to the milking parlour (resulting in cows walking about 8 km/d for twice-daily milking) [31]. Two reports from Australia (who have a smaller national herd size average of about 300 cows [32] compared to New Zealand’s 400 cows [33]) showed that the one-way walking distance to the milking parlour can range from over 2 km [1] to 3.5 km [2]. These reports suggest that a twice-daily milking schedule results in cows walking substantial distances in a single day, and thus may lead to time constraints on the time available for grazing and ruminating in the paddock. Although cows are still able to ruminate while away from the paddock (e.g., while waiting to be milked or during milking), most rumination occurs at night and while lying down [17], so it is important to limit the time away from the paddock. 

On the days when cows spent more time away from the paddock, there was a decrease in lying time and a decrease in milk yield for one of the groups. This result was similar to Beggs et al. [2], who demonstrated that a longer waiting time to be milked negatively impacted lying time in the paddock. Less lying time was also observed in indoor-housed cows when the time out of the pen for milking increased [34,35]. We did not observe a change in grazing or ruminating times on days that the cows spent more time away from the paddock, suggesting that the cows appeared to sacrifice their lying time rather than their grazing or ruminating times when there was less available time in the paddock. The group sizes of this study were small (29 cows each), and the groups were brought to the parlour just before milking, so the waiting time at the parlour was short (i.e., maximum 15 min) and similar across the groups and days. In contrast, pasture-based commercial herds typically experience longer waiting time in the yards (e.g., approximately 3 h (maximum 8 h/d) across two milkings reported on 10 Australian farms with over 500 cows [2]) and a longer total time away from the paddock (e.g., approximately 3.5 to 4 h/d, as self-reported by farmers with herds over 300 cows [1]). Thus, the decrease in lying time observed in our study may underestimate what would be observed in herds of larger sizes that experience more time away from the paddock.

Decreased lying time is frequently cited as an indicator of compromised welfare. Lying provides the opportunity for important rest time, and cows are strongly motivated to lie down (see the review by Tucker et al. [36]). In our study, it appears that the cows made a trade-off to graze for longer and lie down for less time after experiencing more time off-paddock and longer walking distances. This trade-off may not be a welfare concern, so long as cows are able to meet their needs to perform their typical repertoire of behaviours in the paddock (such as eating, ruminating, lying, grooming and social behaviours). On average, the cows in our study spent about 9 h lying, 8 h grazing and 3 h away from the paddock each day, leaving several hours still available in the day to perform other behaviours (some of which was spent ruminating while standing); thus, it is likely that the average cow in our study was not experiencing time constraints to perform grazing, ruminating or lying.

However, some cows in our groups were spending up to 3 h/d more than the average cow engaged in lying or grazing behaviours, meaning that these cows may require more time in the day than other cows to achieve their behavioural needs. Consideration should be given to when behavioural needs are not met, as biological health and functioning could become affected. For instance, a lower lying time (and, thus, greater time spent standing) after calving was a risk factor for claw horn lesions in grazing primiparous cows [37], and a lower lying time between d 20 and 120 postpartum was a risk factor for hoof lesions in indoor-housed cows [38]. Furthermore, milk production can be reduced in cows experiencing lying time restrictions [4], which may relate to reduced blood flow to the udder [39] or reduced plasma growth hormone [40]. This supports our observation in one group that had lower milk yields on days when the time off-paddock was the highest; this relationship was only noted in one of our groups, suggesting that some cows may experience reduced productivity when the off-paddock demands are increased, while other cows are less affected. The group that was affected had a lower average daily milk yield over the study period than the other two groups, which may indicate that lower-producing cows are less likely (compared with higher-producing cows) to adjust their behaviours to compensate for changes in the time spent away from the paddock, resulting in a small negative impact on the milk yield. Although no cows were observed to be lame, other health issues (like mastitis) or oestrus events were not systematically recorded, yet these could affect the cows’ behaviour patterns.

This study was a simple investigation on a single farm limited to three replicate groups. The design of the study permitted each group to experience a different walking distance and time spent off-paddock each day of the study, but the size of the research farm limited the range of walking distances (to a maximum of 4 km) and time spent away (to a maximum of 4 h) that could be tested for their effects on their behaviour and milk yields. Although no work has specifically examined an “acceptable” walking distance or time spent off-paddock, we suggest that our distances and times did not result in a time constraint experienced by all the cows. While our results were limited to small herds of dairy cows, the automated data collection of all the measures recorded in this study provide an opportunity to test if our results are generalizable to the average New Zealand commercial dairy farm.

## 5. Conclusions

Pasture-based dairy cattle are often required to walk several kilometres for milking and they may spend several hours away from the paddock, possibly resulting in time constraints to meet behavioural needs. We found that cows spent more time grazing and less time ruminating on days they walked longer distances (up to 4 km/d) and spent less time lying on days with more time away from the paddock (up to 4 h/d). Our study suggests that cows could benefit from limiting the time away from the paddock and from shorter walking distances; further work is required to determine if these results from small herd sizes are generalizable to typical commercial farms in New Zealand; cows on such farms would be expected to experience a time constraint due to greater walking and time off-paddock demands than those found in this study. 

## Figures and Tables

**Table 1:** Daily behavioural patterns (mean, SD and range (minimum and maximum)) for each of the three groups (29 cows each) managed on a single farm over the experimental period of 13 d. Grazing, ruminating and lying times were measured using accelerometers attached to each cow (data from 29 cows/group). There were no differences in the duration (*p* > 0.1) between the groups for any of the behaviours.

Variable	Mean	SD	Range
**Grazing time (h/d)**			
Group 1	7.5	1.4	4.1–10.0
Group 2	7.7	1.3	3.8–9.6
Group 3	7.6	1.8	3.8–10.1
**Ruminating time (h/d)**			
Group 1	8.2	0.8	6.7–9.4
Group 2	8.4	0.7	7.1–9.8
Group 3	8.1	0.7	7.0–9.3
**Lying time (h/d)**			
Group 1	9.0	0.8	7.2–10.1
Group 2	8.8	0.9	6.9–11.8
Group 3	8.9	1.0	7.4–11.5

## Data Availability

The data can be found in the Mendeley repository at: doi:10.17632/r8bzy85p72.1.
